# Effects of Character Positional Frequency in Chinese Silent and Oral Reading: Evidence from Eye Movements

**DOI:** 10.3390/bs16101719

**Published:** 2026-09-22

**Authors:** Haibo Cao, Zebo Lan, Kuo Zhang, Jingxin Wang

**Affiliations:** 1 College of Education, Hebei University, Baoding 071002, China; psy_haibo@163.com; 2 School of Health, Fujian Medical University, Fuzhou 350122, China; 3 Department of Social Psychology, Zhou Enlai School of Government, Nankai University, Tianjin 300071, China; zhkuo@126.com; 4 Faculty of Psychology, Tianjin Normal University, Tianjin 300380, China; wjxpsy@126.com

**Keywords:** positional frequency, oral reading, silent reading, eye movements

## Abstract

The cognitive mechanisms underlying Chinese word segmentation remain obscure. However, studies have found that readers can use character position probability to facilitate word segmentation, even though the Chinese script does not use spaces. Surprisingly little is known about how this ability is employed during silent and oral reading. The present study manipulated both initial and final character positional frequencies of target words with either high or low lexical frequency. The results revealed a significant reading mode effect, as longer fixations occurred in oral than in silent reading. Critically, a privileged status for initial character positional frequency during word segmentation was observed specifically for low-frequency target words: reliable initial character positional-frequency effects emerged in both silent and oral reading in Experiment 2 but were absent for high-frequency words in Experiment 1. Moreover, the initial character’s positional frequency contributed significantly to the processing of the target word under low-frequency conditions. Taken together, the information on character location frequency is an important clue for readers to segment words, and this processing advantage of the character positional frequency is modulated by lexical word frequency. The findings enhance the development of the character positional decoding model across Chinese reading.

## 1. Introduction

In alphabetic writing systems, inter-word spaces serve as salient visual segmentation cues that support fast and accurate lexical segmentation and word recognition (Bai & Yan, 2017; Li & Pollatsek, 2020). In contrast, written Chinese does not employ inter-word spaces. Readers must therefore rely heavily on intrinsic lexical attributes and statistical regularities to accomplish word segmentation during sentence reading (Cao et al., 2023). One such intrinsic attribute is character-order encoding, which plays a core role in Chinese visual word recognition for neurotypical adults (Gu et al., 2015). For example, the two words 领带 (necktie) and 带领 (lead) share identical constituent characters but differ entirely in semantics solely via character sequence. Developmental-clinical work additionally suggests that dyslexic children exhibit specific deficits in judging character position (Tian et al., 2006). Although these findings have clinical relevance, the present study does not pursue clinical implications and focuses exclusively on neurotypical adult readers. A related statistical regularity, character positional frequency, refers to the statistical tendency of a given morpheme to occur at either the initial or final position of two-character Chinese words (Li et al., 2022; Liang et al., 2023). It is calculated as the frequency with which a character appears in a given word position divided by the total number of two-character compounds that the character can form (Lian et al., 2021). For instance, the character “heng” can form 24 two-character words, among which it occurs word-initially in 21 words, yielding an initial-position positional frequency of 0.88. Repeated exposure to such statistical regularities may shape readers’ positional expectations and produce facilitatory processing effects. According to the integrated model of Chinese reading proposed by Li and Pollatsek (2020), visual-orthographic, sub-lexical morphemic, lexical, and contextual information interact dynamically to control lexical access and eye-movement behavior during Chinese sentence reading. Sub-lexical statistical cues such as character positional frequency constitute one important source of information that can constrain word boundary identification within this theoretical framework.

Empirical evidence has confirmed that character positional frequency functions as a valid implicit statistical cue during Chinese lexical processing. Readers acquire and make use of positional regularities of morphemes to facilitate word segmentation and identification (Lian et al., 2021; Liang et al., 2015). Nevertheless, existing studies yield inconsistent results regarding whether initial-position or final-position positional frequency plays a more dominant role. Some studies highlight the critical function of word-final positional frequency (Liang et al., 2023), whereas other investigations report processing advantages linked to word-initial positional frequency among low-frequency words. These contradictory outcomes suggest that positional-frequency effects are not fixed but modulated by other lexical variables. Among candidate modulating factors, word frequency represents one of the most robust predictors of lexical-processing performance. If word frequency systematically shapes how positional-frequency cues are deployed, it can provide an integrative framework for reconciling prior conflicting results: whether initial- or final-position effects emerge may depend on word-frequency ranges sampled and task properties. Accordingly, testing the modulating role of word frequency aims to uncover the conditional, dynamic nature of positional-frequency effects rather than statically arbitrating which position is intrinsically more important.

According to the augmented addressed-morphology model (Caramazza et al., 1988), lexical access operates via both holistic whole-word routes and morpheme-decomposition routes, whose relative weights depend on lexical familiarity. High-frequency words possess strong whole-word representations and tend to be accessed holistically, which may reduce fine-grained analysis of sub-lexical statistical properties (Cao et al., 2023). Low-frequency words, by contrast, have weaker whole-word representations and rely more heavily on morpheme-level decomposition, rendering morphemic attributes such as positional frequency more consequential for recognition (Cui et al., 2021). Importantly, this account does not claim that morpheme activation disappears entirely for high-frequency words or that whole-word representations are absent for low-frequency words. Instead, word frequency modulates the relative contribution and time course of the two competing processing pathways. Word frequency exhibits consistent effects across languages and reliably indexes cognitive effort during lexical processing (Inhoff & Rayner, 1986; Lian et al., 2026). Taken together, positional-frequency effects should vary dynamically as a function of word frequency: decomposition-based processing dominates for low-frequency words, enabling statistical-cue utilization; holistic whole-word access prevails for high-frequency words and may dampen sensitivity to sub-lexical positional constraints. Word frequency therefore constitutes a promising candidate for resolving existing empirical inconsistencies.

Prior investigations of character positional frequency have largely focused on demonstrating the existence of positional-frequency effects, yet insufficient attention has been paid to explicitly isolating and testing the moderating influence of word frequency. Many previous studies either did not systematically manipulate word frequency or grouped high-frequency and low-frequency items into separate experimental blocks without centering analyses on the conditional interplay between positional-frequency cues and lexical familiarity. As a result, it remains unclear to what extent observed positional-frequency patterns generalize across different word-frequency levels. Beyond lexical-level boundary conditions, another notable area that remains to be further explored concerns reading-mode differences. The overwhelming majority of available empirical data on character positional frequency comes exclusively from silent sentence reading. Oral reading imposes unique cognitive constraints: readers must coordinate eye movements, phonological planning, and overt articulation, producing the well-documented eye–voice span phenomenon (Inhoff et al., 2011; Laubrock & Kliegl, 2015; Lian et al., 2026). Extra coordination demands increase overall fixation durations, yet it remains unclear whether readers still efficiently exploit fine-grained morpheme positional-frequency cues under such load. Recent developmental eye-movement work further demonstrated that lexical variable effects manifest differently across silent versus oral reading contexts (Lian et al., 2026). Two competing viewpoints exist: linguistic segmentation cues operate invariantly regardless of reading mode; alternatively, eye–voice coordination consumes cognitive resources and attenuates the utilization of statistical morpheme-position cues. Resolving these open questions requires an experimental design that orthogonally manipulates positional-frequency properties, explicitly accounts for word-frequency variation, and directly contrasts silent and oral reading.

To resolve these open questions, the present study orthogonally manipulated initial-character positional frequency and final-character positional frequency and compared high- versus low-frequency target words across silent and oral reading using eye-movement recordings. Three a priori hypotheses were formulated. Hypothesis 1: Fixation durations will be reliably longer in oral reading relative to silent reading. Hypothesis 2: Initial-character positional frequency will produce significant facilitatory effects on word processing; two competing predictions exist for final-character positional frequency. If final-character positional frequency provides critical segmentation cues (Liang et al., 2023; Yen et al., 2012), significant facilitatory final-character positional-frequency effects should emerge. Alternatively, if word-initial information is prioritized in Chinese lexical processing (Grainger et al., 2016; Xu & Sui, 2018), final-character positional-frequency effects will be weak or non-significant. Hypothesis 3: Character positional-frequency effects will be more pronounced for low-frequency than for high-frequency target words. Hypothesis 4: There will be a significant interaction between reading mode and character positional-frequency effects. Given the additional phonological-articulatory and eye–voice-coordination burden in oral reading, the facilitatory effects of character positional frequency are expected to be attenuated during oral reading relative to silent reading.

## 2. Experiment 1

### 2.1. Materials and Methods

#### 2.1.1. Participants

Sixty college students were randomly assigned to two between-subject groups (30 silent-reading participants, 30 oral-reading participants). This reading-mode group assignment was kept identical when participants returned for the second experimental session. The participants were 60 college students (20 males and 40 females) aged 18–23 years (*M* = 19.88, *SD* = 0.57). The SIMR package (Version 1.0.2; Green & MacLeod, 2016) for R software (Version 3.3.1; R Development Core Team, 2016) was used to assess power analysis, and based on the effect size in reading times for target words in the Liang et al. (2017) study, it was suggested that the experiment was appropriately powered to detect a character positional-frequency effect (power > 80%). A priori power analysis via SIMR was computed for detecting main effects of character positional frequency, yielding power > 0.80. Note that statistical power for detecting interaction effects is generally lower than for main effects, so our sample may be under-powered for small-to-moderate higher-order interactions.

The participants were native Chinese speakers and had normal or corrected-to-normal vision suitable for reading. Thirty people in each group are under reading oral and silent reading modes. The study was approved by the Research Ethics Committee at the University and conducted in accordance with the principles of the Declaration of Helsinki. We obtained informed consent from participating students before the study was carried out, and each of them was given a monetary reward for participation in the experiment.

#### 2.1.2. Stimuli and Design

Experiment 1 adopted a mixed-factorial design. Reading mode (silent vs. oral reading) served as a between-subject factor; initial-character positional frequency (high vs. low) and final-character positional frequency (high vs. low) were orthogonal within-subject factors. Sixty participants were randomly assigned, 30 for silent reading and 30 for oral reading. Target words in Experiment 1 were high-frequency two-character compound words.

Word frequency was manipulated across two experimental sessions rather than intermixed within a single list. This design choice was driven by severe stimulus-availability constraints: very few high-frequency two-character words satisfy both low-initial- and low-final-character positional-frequency criteria. Intermixing high- and low-frequency targets would lead to unbalanced item numbers and large global sentence-difficulty fluctuations. Separating high- and low-frequency targets into different experimental blocks can be found in existing psycholinguistic studies. Interpretive caveats associated with this block-separated manipulation are acknowledged in the General Discussion.

Word frequency values were extracted from the SUBTLEX-CH corpus (Cai & Byrsbert, 2010), unit: occurrences per million words. Following standard practice in Chinese eye-movement reading research (G. L. Yan et al., 2006; Lian et al., 2026), high-frequency two-character words were defined as >30 occurrences per million, and low-frequency two-character words were defined as <5 occurrences per million. Target words of Experiment 1 satisfied the high-frequency criterion; target words of Experiment 2 satisfied the low-frequency criterion. An independent-samples *t*-test confirmed that word frequency differed significantly between Experiment 1 and Experiment 2 target items, *t*(126) = −13.08, *p* < 0.001.

To obtain appropriate target words, the character positional frequency was computed from the SUBTLEX-CH corpus (Cai & Byrsbert, 2010): the number of characters that occur at the beginning or end of a word divided by the number of two-character words that the character can form (Liang et al., 2017). To differentiate the probability of morpheme location, high character positional frequency was defined as more than 0.7 and low character positional frequency as less than 0.3. Each target word followed four types of positional-frequency conditions (each condition contained 32 two-character words): (1) H-H: a character with high word-beginning and high word-ending positional frequency; (2) H-L: a character with high word-beginning and low word-ending positional frequency; (3) L-H: a character with low word-beginning and high word-ending positional frequency; and (4) L-L: a character with low word-beginning and low word-ending positional frequency.

Additionally, we controlled the strokes and character frequencies of the target words across the four conditions, and no significant differences were obtained: no significant difference was found for word frequency (*F* (3, 124) = 0.51, *p* > 0.05), the stroke number of the initial character (*F* (3, 124) = 1.59, *p* > 0.05), the stroke number of the final character (*F* (3, 124) = 1.91, *p* > 0.05), the frequency of initial characters (*F* (3, 124) = 1.52, *p* > 0.05), or the frequency of final characters (*F* (3, 124) = 0.84, *p* > 0.05). Furthermore, 20 participants who had not participated in the formal experiment were recruited to assess the familiarity ratings of the target words (1 = very unfamiliar, 5 = very familiar, with a higher range representing higher word familiarity). As seen in Table 1, the test presented an effective measure of the familiarity rate (*M* = 4.12, *SD* = 0.19). In addition, four types of target words were manipulated to produce orthographic neighborhood sizes: *M* = 41, 36, 34, and 37, and there were no statistically significant differences (*F* (3, 124) = 0.39, *p* > 0.05).

The sentence stimuli comprised 32 sets of sentences, four types of the target words with the same sentence structures (an example is presented in Table 2). The critical target words were not placed at the beginning of a sentence nor at the end of a sentence. Each sentence contained 18 to 22 characters (*M* = 20.14 characters, *SD* = 0.28) and had the selected two-character target words in the middle. All stimulus materials were arranged for a cloze procedure to test their predictability. An additional 20 participants were asked to write a word that ensured the sentences’ integrity before the target word. Cloze predictability for target words was low, *M* = 0.072 (7.2%), *SD* = 0.25. Condition-wise cloze probability means H-H = 6.8%, H-L = 7.7%, L-H = 7.1%, and L-L = 7.3%. A one-way ANOVA confirmed no significant predictability differences among four positional-frequency conditions, *F* (3, 124) = 0.21, *p* > 0.05.

A 5-point scale (1 = very unnatural/very difficult to understand, 5 = very natural/very easy to understand) was used to rate the naturalness and difficulty of each sentence by an additional 20 participants. Looking at the naturalness (*M* = 4.09, *SD* = 0.23) and difficulty (*M* = 4.11, *SD* = 0.38) scores, the experimental materials were natural and easy for the readers to comprehend.

The sentences were split into four different lists. Each list contained four practice sentences, 32 experimental sentences, and 32 filler sentences (all plausible to minimize participants’ awareness of the experimental purpose). The 32 experimental sentences comprised four different conditions with eight sentences per condition. Using a Latin square, sentences were evenly distributed across lists to ensure each list contained one version per condition while keeping equal condition representation per list. Experimental and filler sentences were displayed in randomized order.

#### 2.1.3. Apparatus and Procedure

Following simple instructions for the experiment, the right eye of the college student was captured by an Eyelink 1000 Plus eye tracker (SR Research Ltd., Ottawa, ON, Canada). The sentences were displayed on a 24-inch Benq LCD monitor (BenQ Corporation, Suzhou, China) with black characters on a white background, and the screen had a resolution of 1920 × 1080 pixels. Each character was 41 × 41 pixels. The participants’ eyes were 70 cm from the screen, and each character subtended approximately 0.9°. The participant’s head was fixed by a chin rest. A 3-point horizontal procedure was used to calibrate the eye tracker (the calibration error was limited to 0.2), and a ten-trial cycle was used for calibration for the main experiment. A cross-shaped sign was placed on the monitor on the left side before the experimental sentence was presented. Whenever the cross-shaped sign was fixated upon, it was replaced by a character, and the sentence was shown on the screen. The participants’ comprehension of sentences was recorded at the touch of a button, and some of the sentences (25%) were followed by a yes/no question based on the textual content, to which the participants pressed appropriate keys to answer. The experiment lasted approximately 20 min.

#### 2.1.4. Analysis

We fitted linear mixed-effects models using the lme4/lmerTest packages in R. Fixed factors included reading mode (between-subject), initial-character positional frequency, final-character positional frequency, and all two-way and three-way interactions. Predictors were sum-to-zero deviation coded via the contr.sdif function. We followed the maximal-random-effects principle (Barr et al., 2013): the starting model contained random intercepts for participants and items, plus random slopes for within-subject factors (initial positional frequency, final positional frequency) over participants and items. When maximal models showed convergence failures, we sequentially removed the highest-order random-slope terms until convergence was achieved. The full formula template: log(DV) ~ reading_mode * initial_pos * final_pos + (1 + initial_pos + final_pos | subject) + (1 + initial_pos + final_pos | item). Convergence warnings were documented; simplified converged random-effect structures were adopted where necessary. Dependent variables (FFD, GD, TRT) were log-transformed prior to modeling, consistent with standard eye-movement analysis practice (Thierfelder et al., 2020; G. Yan et al., 2021).

### 2.2. Results

The average response accuracy on the comprehension questions was 92%, indicating that participants had a relatively accurate understanding of the sentences. Data from the eye-movement measures followed strictly standard procedures, and fixations on the target word with durations <80 ms or >1200 ms were excluded. Trials with tracker loss and those with fewer than five fixations were removed (2.6% of total data). Standard oculomotor parameters were obtained (Rayner, 2009), including first-fixation duration (FFD, the length of the first fixation on the target word), gaze duration (GD, the sum of all first-pass fixations on the target word before the eyes moved to other words), and total reading time (TRT, the sum of fixation times on the target word). The eye-movement measures were analyzed by fitting linear mixed-effects models (Baayen et al., 2008), and the lmer function from the lme4 package (Bates et al., 2015) in R (R Development Core Team, 2016) was applied. Log-transformation of continuous variables was carried out (Baayen et al., 2008). The maximal random-effects model (Barr et al., 2013) was constructed in which the initial and final character positional frequencies were fixed factors, and participants and items were random factors. The contr.sdif function from the MASS package was employed to examine the contrasts of main effects and interaction contrasts (Ripley et al., 2015). The data from silent and oral reading were analyzed and are shown in Table 3 and Table 4.

For every measure, the main effect of reading mode was observed (*t*s > 1.96, *p*s < 0.01): fixation times were longer for oral reading than for silent reading, first fixation duration (*b* = 0.14, *SE* = 0.02, *t* = 5.42, *p* < 0.001), gaze duration (*b* = 0.25, *SE* = 0.04, *t* = 6.91, *p* < 0.001), and total reading time (*b* = 0.16, *SE* = 0.06, *t* = 2.85, *p* < 0.001). The main effects of the initial and final character positional frequency did not reach significance (FFD, GD, TRT, *t*s < 1.96, *p*s > 0.05). No significant interaction between the initial and final character positional frequencies, or a three-way interaction with the reading mode, was found (*t*s < 1.96, *p*s > 0.05). Overall, first-pass target viewing did not vary systematically with character positional frequency. The results suggest that character positional probability did not affect the readers’ eye-movement measures for the high-frequency target words.

### 2.3. Discussion

In Experiment 1, the processing advantage of character positional frequency was not found in silent and oral reading. The variation in the positional frequency of constituent characters had no observable effect on the fixation durations of critical target words. The results, therefore, reflected that word frequency might affect the role of character positional probability. Based on previous studies, high-frequency words are relatively easy to recognize (Blythe et al., 2009; Inhoff & Rayner, 1986) and are accessed as a single entity during cognitive processing. In this situation, the feature of morpheme location probability may be concealed. A noteworthy issue is raised at this point: how does this linguistic statistical attribute behave when the word frequency decreases? The fact is that lower-frequency words are more difficult to decode and are likely to be perceived via decomposed-morphological representation (Peng et al., 1999; G. L. Yan et al., 2006); thus, the probability of character position might have an opportunity to emerge. Hence, we anticipated that readers might benefit from the manipulation of character positional frequency with low-frequency words. Importantly, exploring this issue will help to clarify the influence of lexical frequency on morpheme characteristics.

## 3. Experiment 2

### 3.1. Materials and Methods

#### 3.1.1. Participants

All 60 participants who completed the first experimental session took part in the second experiment. Experimental-sequence counterbalancing was implemented: 30 participants completed Experiment 1 (high-frequency words) first, then Experiment 2 (low-frequency words); the remaining 30 participants completed Experiment 2 first, then Experiment 1. A standardized 30 min rest break was implemented between the two sessions for every participant to alleviate fatigue. The stimulus materials used in Experiment 1 and Experiment 2 were completely distinct, with no overlapping target words or filler sentences, which reduced confounding effects induced by item familiarity. Reading-mode between-subject assignment was unchanged for each participant across both sessions.

#### 3.1.2. Stimuli and Design

Experiment 2 retained exactly the same mixed-factorial design: reading mode remained between-subject; initial- and final-character positional frequency were within-subject factors. All 60 participants took part in Experiment 2, retaining their original reading-mode assignment (silent-reading participants stayed in silent reading; oral-reading participants stayed in oral reading). Target words in Experiment 2 were low-frequency two-character compound words.

The material selection for Experiment 2 was equivalent to that of Experiment 1. Four types of two-character words were chosen (each condition contained 32 two-character words). In the same way, the number of strokes and character frequencies of the target words were controlled: there was no significant difference in word frequency (*F* (3, 124) = 1.35, *p* > 0.05), the stroke number of the initial character (*F* (3, 124) = 0.98, *p* > 0.05), the stroke number of the final character (*F* (3, 124) = 0.97, *p* > 0.05), the frequency of initial characters (*F* (3, 124) = 1.42, *p* > 0.05), or the frequency of final characters (*F* (3, 124) = 1.39, *p* > 0.05). Specifically, the word frequencies of the target words were significantly lower than those in Experiment 1 (*t* = −13.08, *p* < 0.001). Moreover, to obtain appropriate stimuli, 20 participants who had not participated in the formal experiment assessed the familiarity ratings (1 = very unfamiliar, 5 = very familiar, with the higher range representing higher word familiarity) of the target words, and the mean familiarity rating was 3.97 (*SD* = 0.16). Table 5 summarizes the target word characteristics, and the four types of target words with orthographic neighborhood sizes (*M* = 34, 27, 31, 29) showed no significant difference (*F* (3, 124) = 0.42, *p* > 0.05).

The sentence stimuli comprised 32 sets of sentences, four types of the target words with the same sentence structures (an example is presented in Table 6). The critical target words were not placed at the beginning of a sentence nor at the end of a sentence, and each sentence contained 18 to 22 characters (*M* = 20.05 characters, *SD* = 0.32). A cloze procedure was used to evaluate the context predictability of the selected materials, and the participants were asked to write a word to make the sentence meaning complete before the target word. Cloze predictability *M* = 0.043 (4.3%), *SD* = 0.22. Condition-wise cloze probability means H-H = 4.1%, H-L = 4.8%, L-H = 3.9%, and L-L = 4.4%. One-way ANOVA showed no significant differences across four positional-frequency conditions, *F* (3, 124) = 0.16, *p* > 0.05.

The average length of the experimental sentences was 19.88 (*SD* = 0.49). Furthermore, an additional 20 participants were recruited to assess the sentences’ naturalness and difficulty on a 5-point scale (1 = very unnatural/very difficult to understand, 5 = very natural/very easy to understand). The reading materials were quite natural (*M* = 4.03, *SD* = 0.28) and easy (*M* = 3.98, *SD* = 0.16) to comprehend. The design of the two experiments was consistent.

#### 3.1.3. Apparatus and Procedure

The procedure of Experiment 2 was the same as that of Experiment 1.

### 3.2. Results

The accuracy of the participants’ answers to the questions (*M* = 91%) indicated that they read the sentences carefully. The data analysis method was the same as that of Experiment 1 (approximately 2.8% of the data was discarded). Table 7 shows the mean data, and Table 8 summarizes the statistical effects.

Mean gaze-duration and total-reading-time values across conditions are summarized in Table 7 and visualized in Figure 1 and Figure 2. Figure 1 illustrates gaze duration under four experimental conditions for silent and oral reading, with error bars representing standard errors. Figure 2 depicts total reading time across the four conditions in two reading modes.

The viewing times were significantly longer in oral reading than in silent reading across all eye-movement measures: first fixation duration (*b* = 0.12, *SE* = 0.03, *t* = 4.66, *p* < 0.001), gaze duration (*b* = 0.35, *SE* = 0.05, *t* = 6.95, *p* < 0.001), and total reading time (*b* = 0.25, *SE* = 0.06, *t* = 4.07, *p* < 0.001). More specifically, a significant effect of initial character positional frequency emerged, such that the gaze duration (*b* = 0.07, *SE* = 0.03, *t* = 2.39, *p* = 0.02) and total reading time (*b* = 0.13, *SE* = 0.03, *t* = 3.78, *p* < 0.001) were longer for characters with a low initial character positional frequency than for those with a high initial character positional frequency. The main effects of the final character positional frequency did not reach significance (FFD, GD, TRT, *t*s < 1.96, *p*s > 0.05). No significant interaction between the initial and final character positional frequencies, or a three-way interaction with the reading mode, was found (*t*s < 1.96, *p*s > 0.05).

Simple-effect results for Experiment 2: Within silent-reading subset: Gaze duration: *b* = 0.06, *SE* = 0.04, *t* = 1.70, *p* = 0.089; total reading time: *b* = 0.11, *SE* = 0.05, *t* = 2.23, *p* = 0.026.

Within the oral-reading subset: Gaze duration: *b* = 0.08, *SE* = 0.04, *t* = 1.74, *p* = 0.082; total reading time: *b* = 0.14, *SE* = 0.05, *t* = 2.81, *p* = 0.005. The initial-character positional-frequency main effect was significant, and the reading-mode-by-initial-positional-frequency interaction was non-significant, indicating that the magnitude of the initial-positional-frequency facilitation did not reliably differ between silent and oral reading. Follow-up simple-effect analyses showed that initial-positional-frequency effects were visible within each reading-mode subgroup, though some early-time-course gaze-duration comparisons only reached marginal significance. In summary, the findings showed that the initial character position enjoys a very privileged status in Chinese word segmentation. Impressively, readers can use segmentation cues, such as the utility of morpheme-position probability, to facilitate word recognition in both reading modes.

### 3.3. Discussion

In Experiment 2, the results revealed the dominant role of the word-initial character position across the two reading modes. That is, an increase in the initial character positional frequency was accompanied by a reduction in viewing times, whereas no variation occurred at the final character positional frequency. Specifically, the effect of initial character positional frequency was observed throughout both early viewing measures (i.e., gaze durations) and later viewing measures (i.e., total reading times). Due to the lack of visual word boundaries in Chinese text, a character that appears more frequently at the beginning of words can be used to determine which two adjacent characters constitute a word. In addition, word frequency might change readers’ sensitivity to the extraction of positional probability information. Unlike the absence of the effect of character positional frequency in Experiment 1, the present study revealed a preferential role of the word-initial positional frequency under low-frequency conditions. Morpheme decomposition tends to occur in the processing of low-frequency words (Taft et al., 1994; G. L. Yan et al., 2006), which may be conducive to the emergence of the probability feature of morpheme location.

## 4. General Discussion

### 4.1. Differences in Reading Modes

The current research explores the role of character location probability from the perspective of different reading modes and reveals that character positional frequency plays an important role in silent and oral reading. The reading mode affects the results in the form of more reading times for the target words when reading aloud. Owing to the demands of the reading form, in oral reading, every word undergoes the process of pronunciation, no matter whether the word plays a role in understanding the meaning of the sentence. However, words that do not help understand the meaning of sentences can be skipped during silent reading (Laubrock & Kliegl, 2015). Consequently, the viewing times during oral reading were substantially longer than those during silent reading. Furthermore, the process of pronouncing each word in oral reading involves not only the vocal cords but also considerable cognitive resources; hence, it is evident that reading times are associated with cognitive effort (Inhoff et al., 2011; Pan et al., 2017).

It is noteworthy that the pronunciation of words and the fixation of words are inconsistent. Research shows that the pronunciation of words lags one to three words behind the current fixation point (Inhoff et al., 2011; Laubrock & Kliegl, 2015), and thus, the reader must constantly coordinate the distance between the eyes and pronunciation. In this case, cognitive resources are spent on eye–voice coordination, and the mental workload increases in word processing to some extent. The synergy between speech production and cognitive control adds additional processing demands for oral reading, although the initial character positional frequency was reduced when reading aloud, and the character positional frequency-by-reading-mode interaction was not significant. Taken together, oral reading involves dealing with speech planning, and adaptive adjustment associated with eye–voice coordination is concurrently required (Inhoff et al., 2004; Vorstius et al., 2014), which may have a detrimental effect on comprehension.

Contrary to the prediction stated in Hypothesis 4, we did not obtain a reliable reading-mode × character-positional-frequency interaction in the present study. One plausible interpretation is that morpheme-positional statistical cues constitute highly automatic, early-operating orthographic-lexical constraints, which remain largely intact even under the cognitive load associated with eye–voice coordination during oral reading. That is, the extra resources consumed by articulatory planning may elevate overall fixation durations globally for all conditions, without selectively dampening the magnitude of morpheme-position statistical facilitation. Additionally, it should be noted that the statistical power to detect higher-order interactions is generally lower than for main-effect comparisons in the current experimental design; thus, we cannot fully rule out small-to-moderate interaction effects that may have gone undetected. Future studies with larger sample sizes may further test whether oral-reading-specific articulatory load modulates positional-frequency-related processing benefits.

### 4.2. Effects of Character Positional Frequency

Looking at the target fixation change trend across the two experiments, the positional probability of characters significantly affected character-to-word assignment. The results showed that the primary driving force behind the reduction in viewing times is the increase in character positional frequency. The Chinese reading model claims that the characters within the perceptual span are synchronously activated (Li & Pollatsek, 2020). Accordingly, when a word is directly fixated upon, the character that constitutes it is perceived, and the character’s position information is also activated over time. The activation magnitude of character positional information is closely related to the occurrence frequency of the character’s within-word position. That is, the more frequently characters appear in a given position in a word, the more effectively they can be activated. For instance, the character “推” (meaning push) usually occurs at the beginning of words, and many words start with it (e.g., “推动” means promote; “推迟” means delay; “推荐” means recommend). In this case, those words share the same initial character, so the position of the character is easier to activate. Since the character is often placed at the beginning of a word when it appears, the reader realizes that this is the beginning of a word. Consequently, the character positional probability within a word offers a potential parsing cue to demarcate word boundaries.

Accordingly, when the character appears in the same place repeatedly in words, the accumulation of character occurrence frequency will strengthen the connection between the character and its position. Hence, the reader forms a position expectation for this character within words. That is, the character occurring at its usual position affects the ease of word identification. In contrast, inconsistent character positions might cause recognition difficulty, i.e., a character is often used at the beginning of a word, but now it is placed at the final position. This situation forces readers to use their limited cognitive resources to deal with the positional conflict and to confirm the degree of correspondence between the actual position and the habitual position. This effect implied that the qualitative difference was derived from difficulties experienced during the processing of words with lower character positional frequency. Ultimately, the character’s familiar position within words affects the ease of word identification, and the viewing times of the target word increase accordingly.

Especially noteworthy are the results that emerged from the effect of initial character positional frequency; specifically, the initial character position was associated with fewer fixations on the target words, independent of the final character positional frequency. This is impressive because the facilitatory effects reveal the privileged status of word-initial characters in word segmentation. Our study revealed reliable facilitatory effects of initial-character positional frequency for low-frequency target words; by contrast, final-character positional frequency failed to produce significant modulations of reading-time eye-movement measures. This pattern of results partially conflicts with prior evidence of word-final character advantages reported in adult Chinese reading (Liang et al., 2023). Several methodological variables could explain these divergent observations, such as differences in participant samples, computational approaches for deriving positional-frequency values from text corpora, the lexical properties of experimental stimuli, reading-task constraints, and the additional phonological-articulatory burden associated with oral reading (Lian et al., 2026).

Empirical studies have provided evidence that the word-initial letter plays a critical role in word recognition (Johnson & Eisler, 2012; Lima & Pollatsek, 1983; Xu & Sui, 2018). This could occur for several possible underlying reasons. The amount of important information carried at the beginning of a word is greater than that carried at other positions within the word (Grainger & Jacobs, 1993; Shillcock et al., 2000; White et al., 2008). Taft (2004) pointed out that the initial morpheme is the main search target during lexical access, and the influence of the final morpheme is attenuated. Moreover, serial left-to-right scanning impacts the ease of word-initial letter processing (Pollatsek et al., 2006; Tydgat & Grainger, 2009), and the initial character of the word has more possible lexical candidates (Clark & O’Regan, 1999) and is easier to integrate with the text. According to the SERIOL model (Whitney, 2001), the activation intensity of a word is closely bound up with the spatial coding of letter nodes, such that the degree of activation decreases gradually from left to right. As a result, word-initial characters achieve more activation and thus yield a significant unique contribution to word processing.

### 4.3. Effect of Word Frequency on Character Positional Frequency

The discrepancy derived from the effect of character positional frequency is closely related to word frequency across the two experiments. In Experiment 1, an attenuated influence of character positional frequency under high-frequency conditions was observed. Interestingly, however, the effect of initial character positional frequency was pronounced in Experiment 2. In general, then, we assume that words with low frequency are more sensitive to character positional frequency. Since the recognition of low-frequency words may be associated with more cognitive effort (Vorstius et al., 2014), parsing the specific attributes contained in the vocabulary may lead to greater susceptibility to the word’s linguistic properties. In other words, low-frequency words tend to be stored as morphemes (White, 2008), so the features of characters are more easily displayed. Consequently, the property of character positional frequency can exert considerable influence on reading and suggest that morpheme-decomposition representation occurs in word processing. Indeed, the existence of the character positional-frequency effect on low-frequency word conditions has been taken to be indicative of the use of a decomposition pathway, while the absence of the character positional-frequency effect on high-frequency word conditions has been taken to be indicative of whole-word access; these findings support the augmented addressed morphology model.

The observed pattern is suggestive and indirectly consistent with predictions derived from the augmented-addressed-morphology dual-route model (Caramazza et al., 1988): low-frequency words may more readily engage morpheme-decomposition processing, making morpheme-positional statistical properties available to constrain word segmentation; high-frequency words may tend toward holistic whole-word access, which could attenuate sensitivity to constituent-character positional-frequency cues. We stress, however, that eye-movement reading-time patterns alone cannot uniquely verify the existence of distinct processing routes. Alternative accounts remain possible, and converging evidence from additional methodologies such as masked priming or ERP would be required for more definitive conclusions.

Contrary to less frequent exposure to the text, e.g., unfamiliar words, high-frequency words frequently appear in silent and oral reading (Vorstius et al., 2014). High-frequency words are processed by direct lexical access (Joseph et al., 2013; Rau et al., 2014) and are preferentially processed automatically enough to allow for decoding and integration holistically. Hence, the reduced relative difficulty of high-frequency words may be effective in reducing the challenge produced by the probability of character position. Compared with high-frequency words, low-frequency words are less exposed to normal text and are identified with more difficulty than high-frequency words (Liu et al., 2020; Monster et al., 2022; Rayner, 2009), and more cognitive resources might be required to use morpheme features to help vocabulary processing. In this case, morphemic decomposition tends to occur, and the properties of character positional probability produce an effective impact on word segmentation.

We observed reliable initial-character positional-frequency effects for low-frequency words (Experiment 2), but no detectable effect for high-frequency words (Experiment 1). We emphasize that simply contrasting significant versus non-significant results across separate experiments cannot statistically establish that these effects reliably differ from each other (Nieuwenhuis et al., 2011). Given distinct stimulus-item pools for high- and low-frequency conditions, multiple lexical properties besides word frequency may differ. Therefore, our pattern is consistent with the theoretical prediction that lexical word frequency modulates positional-frequency utilization, yet definitive causal evidence requires future study where qualified lexical materials permit within-list word-frequency manipulation. The current work offers a novel perspective that word frequency is an important factor affecting the role of morpheme location probability. Clarifying the nature of positional probabilities of characters aids in a comprehensive understanding of the dynamics of Chinese word segmentation.

### 4.4. Limitations and Future Directions

The present study contains several noteworthy methodological and interpretive limitations. First, word frequency was manipulated across separate experimental sessions rather than within a single experimental list. High-frequency and low-frequency target words draw upon non-identical stimulus pools, so lexical properties other than word frequency may differ between the two sets of materials. This design constraint prevents a direct statistical test for the critical word-frequency × initial-positional-frequency interaction. Future work should strive for within-list word-frequency manipulation if sufficient qualified lexical materials can be obtained. Second, although experimental-sequence counterbalancing was implemented, subtle strategic shifts, practice-related effects, or fatigue across two testing sessions cannot be completely ruled out, even with a standardized rest interval and non-overlapping stimulus materials. Separate participant groups for high- versus low-frequency conditions could be adopted in follow-up research to fully eliminate session-related confounds. Third, our sample was restricted to university-age native Chinese adults; results cannot directly generalize to child-developing readers or clinical populations such as dyslexic readers. Developmental eye-movement research (Lian et al., 2026) indicates that statistical cue utilization changes with reading experience, which deserves further investigation. Fourth, we exclusively tested two-character compound words; positional-frequency mechanisms may differ for longer multi-character Chinese words. Finally, eye-movement latency patterns are correlational and cannot constitute definitive proof for distinct whole-word holistic access versus morpheme-decomposition processing routes. Our dual-route-model-related interpretations remain plausible indirect inferences. Converging evidence from ERP or masked-priming paradigms would strengthen theoretical conclusions.

## 5. Conclusions

In summary, the benefit of shorter viewing durations comes with the advantage of high character positional frequency; more than anything, the effect was pronounced in both reading modes. This means that character positional frequency positively impacts silent and oral reading. Furthermore, this pattern of results points to initial character positional frequency playing an important role in Chinese word segmentation and recognition.

## Figures and Tables

**Figure 1:**
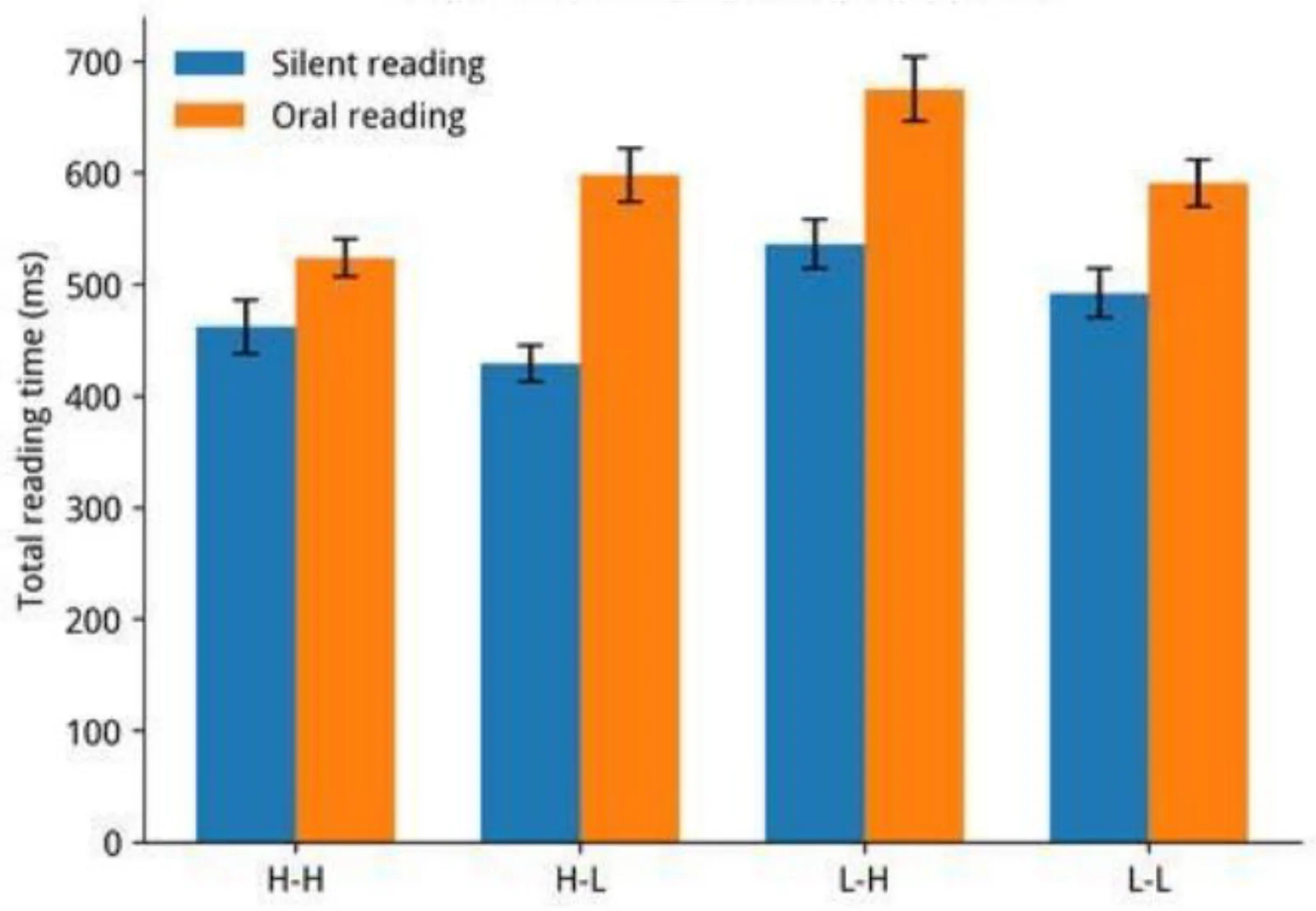
Mean gaze duration (ms) for four conditions (H-H, H-L, L-H, and L-L) in silent reading and oral reading. Error bars represent standard errors.

**Figure 2:**
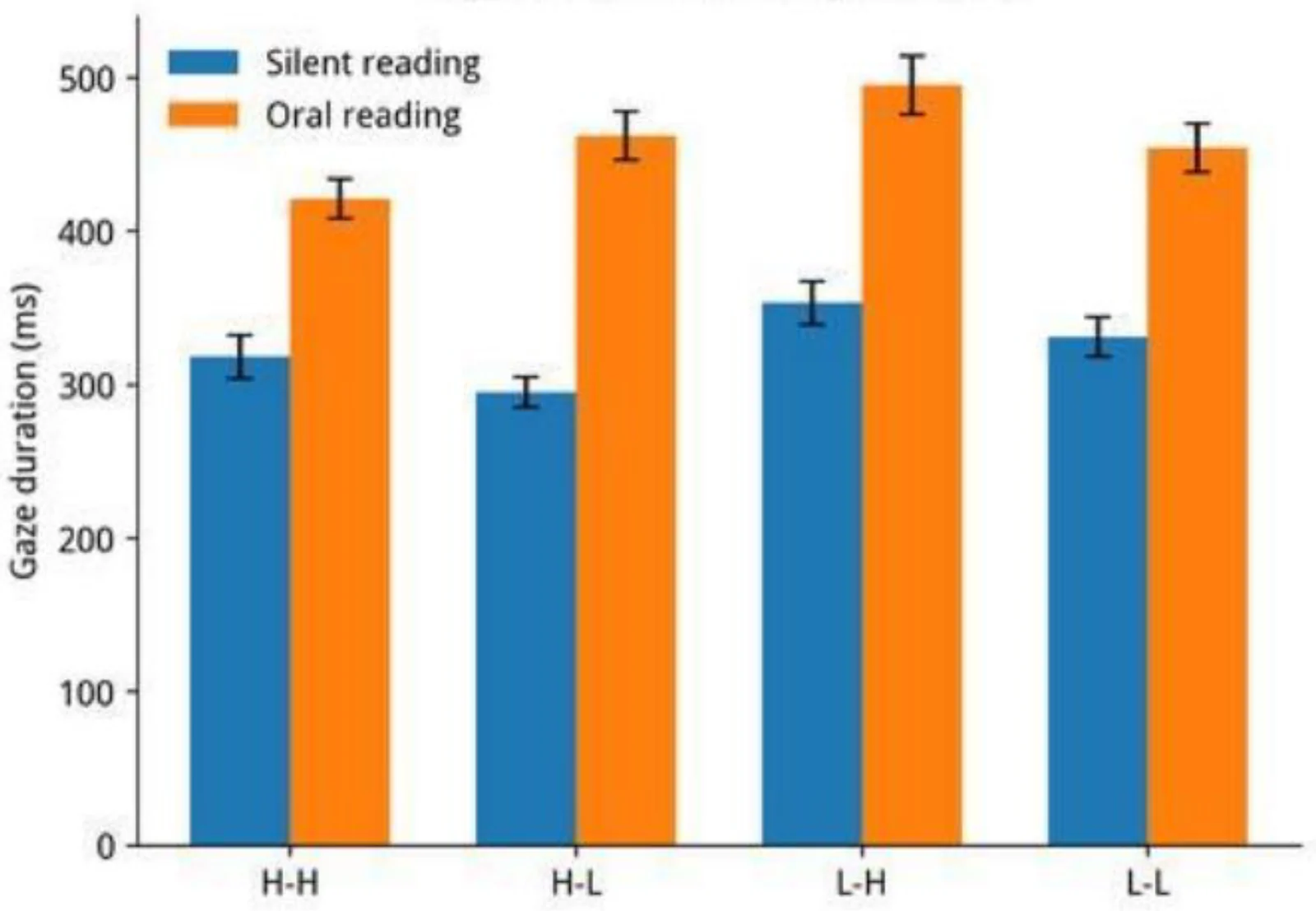
Mean total reading time (ms) for four conditions (H-H, H-L, L-H, and L-L) in silent reading and oral reading. Error bars represent standard errors.

**Table 1:** Descriptive properties of the target in Experiment 1.

Positional Frequency	Initial Positional Frequency	Final Positional Frequency	Word Frequency	Initial Character Stroke	Final Character Stroke	Initial Character Frequency	Final Character Frequency
H-H	0.80 (0.02)	0.78 (0.02)	49.18 (4.55)	9.63 (0.36)	9.66 (0.62)	284.19 (44.58)	376.87 (93.16)
H-L	0.79 (0.02)	0.29 (0.02)	54.89 (8.67)	9.53 (0.35)	8.50 (0.48)	339.35 (93.99)	348.73 (48.62)
L-H	0.25 (0.01)	0.79 (0.02)	50.71 (6.72)	9.28 (0.46)	8.53 (0.47)	335.34 (30.56)	362.12 (35.94)
L-L	0.29 (0.02)	0.35 (0.03)	43.66 (5.34)	8.44 (0.52)	7.88 (0.56)	349.01 (41.92)	295.31 (51.43)

*Note.* H-H = character with high word-beginning and high word-ending positional frequency; H-L = character with high word-beginning and low word-ending positional frequency; L-H = character with low word-beginning and high word-ending positional frequency; L-L = character with low word-beginning and low word-ending positional frequency; the standard error is shown in parentheses. Word/character frequency: 1 million words.

**Table 2:** An example of Chinese sentences in each character positional-frequency condition in Experiment 1.

Conditions	Sentences
H-H	工作人员强调这种**提醒**是很有现实意义的事情。
	The staff emphasized that this kind of **reminder** is very practical.
H-L	工作人员强调这种**预约**是很有现实意义的事情。
	The staff emphasized that this kind of **appointment** is very practical.
L-H	工作人员强调这种**训练**是很有现实意义的事情。
	The staff emphasized that this kind of **training** is very practical.
L-L	工作人员强调这种**尝试**是很有现实意义的事情。
	The staff emphasized that this kind of **attempt** is very practical.

*Note.* The target words are bold but are presented normally during the reading.

**Table 3:** Summary of eye movement measures in Experiment 1.

	Silent Reading				Oral Reading			
Measure	H-H	H-L	L-H	L-L	H-H	H-L	L-H	L-L
First fixation duration (ms)	248 (5)	251 (6)	245 (6)	253 (6)	291 (7)	296 (8)	284 (6)	282 (7)
Gaze duration (ms)	288 (9)	282 (9)	283 (9)	301 (10)	388 (12)	386 (14)	373 (11)	355 (12)
Total reading time (ms)	383 (13)	411 (17)	368 (14)	431 (17)	455 (16)	474 (17)	462 (16)	465 (17)

*Note*. The standard error is shown in parentheses.

**Table 4:** Summary of statistical effects in Experiment 1.

Measure	Effect	*b*	*SE*	*t*	CI
First fixation duration (ms)	Group	0.14	0.02	5.42 ***	[0.09, 0.18]
	Initial	−0.01	0.02	−0.68	[−0.05, 0.03]
	Final	0.01	0.02	0.37	[−0.03, 0.05]
	Group × Initial	−0.02	0.04	−0.52	[−0.10, 0.06]
	Group × Final	−0.01	0.04	−0.14	[−0.09, 0.08]
	Initial × Final	0.00	0.04	−0.05	[−0.08, 0.08]
	Group × Initial × Final	−0.05	0.08	−0.58	[−0.22, 0.12]
Gaze duration (ms)	Group	0.25	0.04	6.91 ***	[0.18, 0.32]
	Initial	−0.02	0.02	−0.74	[−0.06, 0.03]
	Final	−0.02	0.03	−0.63	[−0.06, 0.03]
	Group × Initial	−0.07	0.07	−1.01	[−0.20, 0.06]
	Group × Final	−0.04	0.07	−0.59	[−0.17, 0.09]
	Initial × Final	0.02	0.05	0.33	[−0.08, 0.11]
	Group × Initial × Final	−0.13	0.14	−0.92	[−0.39, 0.14]
Total reading time (ms)	Group	0.16	0.06	2.85 *	[0.05, 0.27]
	Initial	0.00	0.03	0.09	[−0.06, 0.07]
	Final	0.05	0.04	1.31	[−0.02, 0.11]
	Group × Initial	−0.01	0.10	−0.09	[−0.21, 0.19]
	Group × Final	−0.06	0.10	−0.63	[−0.27, 0.14]
	Initial × Final	0.04	0.07	0.56	[−0.10, 0.18]
	Group × Initial × Final	−0.15	0.21	−0.74	[−0.56, 0.25]

*Note.* Group = group of silent and oral reading; Initial = initial character positional frequency; Final = final character positional frequency. * *p* < 0.05 and *** *p* < 0.001.

**Table 5:** Descriptive properties of the target in Experiment 2.

Positional Frequency	Initial Positional Frequency	Final Positional Frequency	Word Frequency	Initial Character Stroke	Final Character Stroke	Initial Character Frequency	Final Character Frequency
H-H	0.82 (0.08)	0.80 (0.08)	2.18 (0.40)	9.56 (1.50)	9.53 (0.46)	180.75 (30.92)	167.84 (32.16)
H-L	0.82 (0.09)	0.22 (0.07)	1.78 (0.38)	9.03 (0.49)	9.66 (0.52)	111.82 (27.85)	101.45 (13.17)
L-H	0.21 (0.06)	0.81 (0.09)	2.38 (0.55)	9.25 (0.38)	9.04 (0.48)	177.50 (26.66)	202.62 (33.69)
L-L	0.25 (0.06)	0.26 (0.06)	1.63 (0.31)	9.00 (0.37)	9.91 (0.49)	266.61 (38.59)	242.51 (52.00)

*Note.* The standard error is shown in parentheses. Word/character frequency: 1 million words.

**Table 6:** An example of Chinese sentences in each character positional-frequency condition in Experiment 2.

Conditions	Sentences
H-H	有一些专家认为**倾销**无助于解决这个问题。
	Some experts believe that **dumping** will not help solve this problem.
H-L	有一些专家认为**偏执**无助于解决这个问题。
	Some experts think **paranoia** will not help solve this problem.
L-H	有一些专家认为**袭扰**无助于解决这个问题。
	Some experts believe that **harassment** will not help solve this problem.
L-L	有一些专家认为**类推**无助于解决这个问题。
	Some experts believe that **analogy** will not help solve this problem.

Note. The target words are bold but are presented normally during the reading.

**Table 7:** Summary of eye movement measures in Experiment 2.

	Silent Reading				Oral Reading			
Measure	H-H	H-L	L-H	L-L	H-H	H-L	L-H	L-L
First fixation duration (ms)	262 (7)	248 (6)	267 (7)	260 (7)	296 (8)	301 (7)	298 (7)	289 (7)
Gaze duration (ms)	318 (14)	295 (10)	353 (14)	331 (13)	421 (13)	462 (16)	495 (19)	454 (16)
Total reading time (ms)	462 (24)	429 (16)	536 (22)	492 (22)	524 (17)	598 (24)	675 (29)	591 (21)

*Note.* The standard error is shown in parentheses.

**Table 8:** Summary of statistical effects in Experiment 2.

Measure	Effect	*b*	*SE*	*t*	CI
First fixation duration (ms)	Group	0.12	0.03	4.66 ***	[0.07, 0.18]
	Initial	0.01	0.02	0.49	[−0.03, 0.05]
	Final	−0.02	0.02	−0.84	[−0.05, 0.02]
	Group × Initial	−0.06	0.04	−1.44	[−0.14, 0.02]
	Group × Final	0.02	0.04	0.62	[−0.05, 0.09]
	Initial × Final	0.00	0.04	0.06	[−0.08, 0.08]
	Group × Initial × Final	−0.06	0.08	−0.79	[−0.21, 0.09]
Gaze duration (ms)	Group	0.35	0.05	6.95 ***	[0.25, 0.45]
	Initial	0.07	0.03	2.39 *	[0.01, 0.14]
	Final	−0.02	0.03	−0.49	[−0.08, 0.05]
	Group × Initial	−0.04	0.06	−0.69	[−0.16, 0.08]
	Group × Final	0.03	0.06	0.54	[−0.09, 0.16]
	Initial × Final	−0.06	0.06	−0.98	[−0.18, 0.06]
	Group × Initial × Final	−0.10	0.12	−0.82	[−0.34, 0.14]
Total reading time (ms)	Group	0.25	0.06	4.07 ***	[0.13, 0.37]
	Initial	0.13	0.03	3.78 **	[0.06, 0.20]
	Final	−0.03	0.04	−0.72	[−0.10, 0.05]
	Group × Initial	−0.05	0.07	−0.69	[−0.18, 0.09]
	Group × Final	0.02	0.07	0.25	[−0.12, 0.16]
	Initial × Final	−0.12	0.07	−1.71	[−0.27, 0.02]
	Group × Initial × Final	−0.07	0.16	−0.45	[−0.38, 0.24]

*Note*. * *p* < 0.05, ** *p* < 0.01, *** *p* < 0.001.

## Data Availability

The datasets and materials generated and analyzed during the current study are available on the Open Science Framework at https://doi.org/10.17605/OSF.IO/23TEJ.
